# Progress in NK cell-based immunotherapy for solid tumors and an exploration of its cardiovascular safety

**DOI:** 10.3389/fcell.2026.1927923

**Published:** 2026-10-16

**Authors:** Jiaxin Chen, Juntong Shen, Xiaolin Fu, Min Jiang, Ruihao Liu, Qiang Qiao, Yizhe Hu

**Affiliations:** Ansteel Group General Hospital, Anshan, China

**Keywords:** cardiovascular safety, CAR-NK, cell-based therapy, NK cell-based immunotherapy, solid tumors

## Abstract

Natural killer (NK) cell-based immunotherapy has become a research focus in cellular immunotherapy in recent years,which has advanced rapidly in the treatment of solid tumors. NK cells are key effector cells of innate immunity and can kill abnormal target cells without prior sensitization. A broad consensus holds that NK cell-based immunotherapy may become a new strategy for solid tumor treatment with lower risk and lower cost than T-cell-based immunotherapy. However, its translation from theory to clinical application remains constrained by relevant risks, and research on its cardiovascular safety is still largely absent. This review summarizes the latest research progress of NK cell-based immunotherapy in solid tumors and discusses its cardiovascular safety.

## 1 Introduction

Solid tumors are tumors with a defined histological structure. According to GLOBOCAN 2022 estimates from the International Agency for Research on Cancer (IARC), approximately 20 million new cancer cases occur worldwide each year, of which solid tumors account for more than 90%; lung cancer, breast cancer, colorectal cancer, prostate cancer, and gastric cancer alone account for 45.8% of global cancer incidence (Bray et al., 2024). Immunotherapy has the potential to elicit durable responses and achieve deep remissions in solid tumors (Tocchetti et al., 2024). ICIs have demonstrated durable responses and deep remissions in selected solid tumors; CAR-T therapy remains investigational for solid tumors (Tocchetti et al., 2024). These strategies have already made significant progress (Tocchetti et al., 2024). In recent years, NK cell-based immunotherapy has attracted substantial attention in the field because of their widely recognized “low-risk” label and potential as “off-the-shelf” products.

Compared with hematologic malignancies, patients with solid tumors exhibit markedly different demographic and clinical characteristics: the median age at onset is higher (lung cancer and gastrointestinal tumors often occur at 60–70 years of age), the burden of cardiovascular comorbidities is heavier (hypertension prevalence 26.0%, coronary heart disease 7.8%, and heart failure 3.3%) (Liu et al., 2020), and prior anthracycline chemotherapy or thoracic radiotherapy often leaves the myocardium in a “pre-injured” state. Studies have shown that immunotherapies such as ICIs and CAR-T can cause cardiovascular toxicity through mechanisms including cytokine release syndrome (CRS), and together with the tumor microenvironment (TME), can aggravate cardiovascular injury. Therefore, as NK cell-based immunotherapy strategies expand from hematologic malignancies into solid tumors, their cardiovascular safety should be comprehensively evaluated and corresponding clinical management standards should be developed. At present, the cardiovascular toxicity of NK cell-based immunotherapy represented by chimeric antigen receptor NK cell therapy (CAR-NK) has not yet been systematically observed or studied. Filling this gap in cardiovascular risk research will be an unavoidable issue in translating NK cell therapy from theory into clinical practice.

This review aims to elucidate the mechanisms underlying NK cell-based therapies, introduce current strategies for enhancing NK cell function in the tumor microenvironment (TME), and focus on the advantages of CAR-NK cells. Integrate the fragmented clinical data on existing CAR-NK therapies, and draw on the established mechanisms of cardiovascular damage associated with other cellular immunotherapies to infer potential cardiovascular risks associated with CAR-NK therapies. Finally, we will discuss how to proceed with future research on clinical theory and practice.

## 2 Chapter 1: mechanisms and advantages of NK cell immunotherapy for solid tumors

### 2.1 NK cells can recognize and kill solid tumor cells

NK cells are key effector cells of the nonspecific immune system and play an indispensable role in immune surveillance and clearance of solid tumors. Unlike T cells, NK cells can recognize and kill abnormal cells without prior sensitization by integrating the balance of signals from activating and inhibitory surface receptors (Campbell and Hasegawa, 2013). The antitumor activity of NK cells depends on the coordinated regulation of multiple molecular pathways: binding of the activating receptor NKG2D to its stress-induced ligands, such as MICA/B or ULBPs, is a key node for initiating cytotoxicity (Wang et al., 2024; Kang, 2025); natural cytotoxicity receptors (NCRs; NKp30, NKp44, and NKp46) recognize tumor-specific antigens; and CD16 (FcγRIIIa) mediates antibody-dependent cellular cytotoxicity (ADCC) (Campbell and Hasegawa, 2013; Wang et al., 2024; Kang, 2025). After recognizing abnormal cells and becoming activated, NK cells mainly induce target-cell death through two pathways: (1) releasing perforin and granzymes to form membrane pores and directly induce tumor-cell apoptosis; (2) triggering extrinsic apoptotic pathways through membrane-bound death ligands such as FasL and TRAIL (Wang et al., 2024; Kang, 2025; Luo et al., 2025). In addition to direct killing, NK cells secrete cytokines such as IFN-γ and TNF-α to activate dendritic cells and T cells and initiate specific immune responses (Campbell and Hasegawa, 2013; Luo et al., 2025). When encountering normal host tissues, NK cells recognize MHC class I molecules on the surface of normal cells through inhibitory receptors such as KIR and NKG2A, thereby generating immune tolerance and avoiding autoimmune injury (Campbell and Hasegawa, 2013; Dizaji Asl et al., 2021).

### 2.2 The physiological effects of NK cells are suppressed in the solid tumor microenvironment

Although NK cells have powerful cytotoxic capacity, their physiological effects are severely suppressed under the influence of the TME, making it difficult for them to exert their full strength. High concentrations of immunosuppressive cytokines in the TME, such as TGF-β and IDO, can directly inhibit NK cell activation and proliferation (Wang et al., 2024). On the other hand, TME is characterized by nutrient deprivation, hypoxia, and the accumulation of metabolic byproducts (Wang et al., 2025a; Li et al., 2026). During this process, tumor cells deprive immune cells of the nutrients they need and produce immunosuppressive substances such as lactate and adenosine (Wang et al., 2025a; Li et al., 2026). In environments characterized by nutrient deprivation and hypoxia, tumor cells can adapt metabolically through other pathways, such as fatty acid oxidation and glutamine breakdown (Wang et al., 2025a). Accumulation of metabolites such as adenosine and prostaglandin E2 can further weaken NK cell cytotoxicity (Wang et al., 2024; Luo et al., 2025). The upregulation of HIF-1α commonly observed in solid tumors can induce mitochondrial dysfunction in NK cells and inhibit aerobic glycolysis, causing NK cell energy-metabolism failure (Luo et al., 2025) and making it difficult to sustain efficient effector function. At the same time, abnormal expression of surface molecules on NK cells in the TME also contributes to suppression of their cytotoxic effects: NK cells in the TME highly express inhibitory receptors such as PD-1, TIM-3, LAG-3, and TIGIT, which transmit negative signals into NK cells after binding their ligands and inhibit NK cell killing function (Wang et al., 2024; Luo et al., 2025). In single-cell atlases of breast cancer and hepatocellular carcinoma, NK cell subsets with specific inhibitory genes (such as high expression of the NR4A family and KLRC1) show obvious functional exhaustion phenotypes, and the corresponding patients often have relatively poor prognoses (Xu et al., 2024; Huang Y. et al., 2025). In summary, the TME blocks the cytotoxic effects of NK cells through multidimensional mechanisms including metabolic deprivation, upregulation of inhibitory ligands, and cellular-subset reprogramming. Restoring the physiological functions of NK cells in the TME, or targeting these pathways therapeutically, may be an effective approach for treating solid tumors (Wang et al., 2024; Xu et al., 2024). It has also made NK cell-based immunotherapy a major focus in recent years.

### 2.3 Therapeutic strategies for restoring NK cell activity

Based on the mechanisms by which the TME suppresses NK cell effects, three major directions can be considered to reverse TME-induced NK cell exhaustion and restore NK cell activity: genetic engineering, antibody-mediated activation, and cytokine/metabolic regulation.

1. Genetic engineering strategy: CAR-NK.

CAR-NK refers to genetically engineered NK cells that express chimeric antigen receptors (CARs) capable of specifically recognizing tumor antigens, thereby enhancing precise tumor killing. Compared with CAR-T, CAR-NK carries a lower risk of severe CRS and has fewer adverse effects (Peng et al., 2024; Pan et al., 2022). With advances in genetic engineering, genome-wide CRISPR screening has identified negative regulators such as MED12, ARIH2, and CCNC. Knockout of these genes can significantly enhance the metabolic adaptability and antitumor activity of NK cells (Biederstädt et al., 2025). Chemokine receptors such as CXCR4 can also be introduced to improve tumor infiltration (Ran et al., 2022). Existing preclinical evidence shows that CAR-Tcells targeting antigens such as HER2 are effective in solid tumor models to effectively kill neighboring tumor cells. It may be used in CAR-NK engineering (Pascual-Pasto et al., 2024; Maalej et al., 2023).

2. Antibody-mediated strategies to enhance NK cell function.

Bispecific and trispecific immune-cell engagers (BsAbs/TsAbs) induce immune-synapse formation by bridging tumor antigens and NK cell activating receptors such as CD16 (Tapia-Galisteo et al., 2023). Novel trispecific nanobodies, such as those targeting PD-L1, 4-1BB, and NKG2A, can synergistically activate NK cells and CD8^+^ T cells, significantly inhibit tumor growth in solid tumor models, and prolong survival of tumour - bearing mice (Ye Q.-N. et al., 2024). Blocking inhibitory checkpoints such as PD-1/PD-L1 and TIGIT can restore NK cell effector function and shows synergistic effects when combined with CAR therapy (Maalej et al., 2023; Laskowski et al., 2022). Fc-engineered antibodies further enhance ADCC by optimizing binding affinity to CD16 (Tapia-Galisteo et al., 2023).

3. Cytokine and metabolic regulatory strategies.

Regulation of cytokines in the microenvironment is also a key means of restoring NK cell activity. IL-15 superagonists and receptor complexes, such as IL-15/IL-15Rα-Fc, can maintain NK cell proliferation and physiological activity (Laskowski et al., 2022). Novel surrogate cytokine agonists can use bispecific ligands to induce non-natural receptor dimerization, enabling precise shaping of signaling pathways, and some products show potent antiviral and immune-activating activity (Yen et al., 2022). In terms of metabolism, intermittent fasting promotes glucocorticoid secretion, induces CPT1A expression, and promotes fatty acid oxidation (FAO), allowing NK cells to maintain survival, proliferation, and physiological effects under nutrient-poor conditions in the TME (Delconte et al., 2024). CRISPR screening has also confirmed that regulating metabolism-related genes, such as the mTORC1 pathway, can enhance NK cell stress resistance in the TME (Biederstädt et al., 2025).

### 2.4 NK cell-based immunotherapy has multidimensional advantages

Compared with T-cell-based immunotherapy, NK cell-based immunotherapy demonstrates advantages across multiple dimensions.

NK cells recognize abnormal cells through MHC-I loss (Dagher and Posey, 2023) and do not need to anchor to a single abnormal antigen before exerting cytotoxic effects as T cells do. MHC-I downregulation caused by genomic instability is common of solid tumors, giving NK cells a natural advantage in recognizing tumor cells (Dizaji Asl et al., 2021; Peng et al., 2024): NK cells can bypass the time-consuming steps of antigen sensitization and human leukocyte antigen (HLA)-restricted presentation, and they also possess broad defensive capacity against resistance caused by antigen-loss variants (LOH) or target downregulation (Pan et al., 2022). Because of their low risk of graft-versus-host disease (GvHD) and lack of a requirement for HLA matching, NK cells have the potential to become true “off-the-shelf” products (Maalej et al., 2023). CAR-T requires an individualized and lengthy manufacturing process of patient blood collection, *ex vivo* activation, viral transduction, expansion, and reinfusion, usually taking at least 3 weeks (Pu et al., 2024). Compared with CAR-T, NK cells can be derived from cord blood, peripheral blood, induced pluripotent stem cells (iPSCs), or immortalized NK-92 cell lines, enabling standardized and scaled production with strict quality control (Pu et al., 2024). This greatly shortens the waiting time for treatment while significantly reducing manufacturing costs and operational complexity. The factors mentioned above give CAR-NK therapy significant advantages. In addition, NK cells show stronger tolerance than T cells within the TME. NK cells naturally highly express chemokine receptors such as CXCR3, CXCR4, and CCR5, enabling them to respond effectively to chemokine signals such as CXCL9/10/12 and CCL5 secreted by the tumor stroma and to achieve directed infiltration (Peng et al., 2024; Maalej et al., 2023).

At the cytokine-network level, NK cells also have unique advantages. Their endogenous secretion of interferon-γ (IFN-γ) can not only directly inhibit tumor proliferation but also reprogram tumor-associated macrophages (TAMs), shifting them from a tumor-promoting M2 phenotype to an antitumor M1 phenotype and reversing the immunosuppressive microenvironment (Dagher and Posey, 2023). When combined with IL-15 superagonists such as ALT-803 or N-803, NK cells can efficiently induce the generation of memory-like NK cells, a subset that survives persistently *in vivo*, reactivates rapidly, and has stronger sustained killing capacity (Pu et al., 2024). In energy metabolism, NK cells rely more on oxidative phosphorylation than glycolysis compared with T cells; they have higher mitochondrial quality and stronger fusion, giving them better energy-use efficiency and fatigue resistance in the TME (Dagher and Posey, 2023).

CAR-NK also has safety advantages: the incidence of CRS and neurotoxicity is extremely low, and no definitive reports of grade ≥3 CRS or immune effector cell-associated neurotoxicity syndrome (ICANS) have been observed to date (Maalej et al., 2023; Pu et al., 2024; Farmanieh et al., 2026). Allogeneic NK cell infusion also almost never induces GvHD: NK cells do not express T-cell receptors (TCRs), and their cytotoxic activity is regulated by KIR-HLA ligand interactions, resulting in a lower risk of attacking recipient tissues (Dizaji Asl et al., 2021; Pu et al., 2024). This gives NK cells a high level of safety even in immunodeficient hosts and broadens the applicable patient population.

## 3 Chapter 2: the generality and mechanistic features of cardiotoxicity in solid tumor immunotherapy

### 3.1 Immunotherapy can cause multiple cardiac adverse events

Immunotherapy-related cardiotoxicity (ICIs-CTRCD) is gradually being recognized as a class of “effect-like cardiovascular complications” with a clear pathological basis, an observable clinical spectrum, and broad relevance. Immune-mediated inflammatory injury shows highly heterogeneous clinical phenotypes: fulminant myocarditis, such as pembrolizumab-induced cases with a sudden drop in LVEF to <10% requiring ECLS support (Nguyen et al., 2022); subacute left ventricular dysfunction, with 12%–28% of patients developing mostly reversible LVEF decline after CAR-T (Totzeck et al., 2022); arrhythmias, including atrial fibrillation, ventricular tachycardia, and conduction block, with acombined incidence of 11.5% (Liu Y. et al., 2025); vasculitis-like manifestations such as venous thromboembolism and capillary leak; and pericardial disease (2.0%) (Totzeck et al., 2022). The overall incidence of ICI-related cardiotoxicity is about 1%–2%, with a fatality rate of about up to 46% (Munir et al., 2026), significantly higher than myocardial injury caused by conventional chemotherapy (Patone et al., 2022). In CAR-T treatment, cardiovascular adverse events (CPAEs) occur in 20.5% of patients, among which hypotension (52.8%) and tachycardia (the incidence rate among pediatric patients was 69.2%, while that among adult patients was 12.2%) present as hyperacute events with a median onset of 6–21 days (Totzeck et al., 2022; Joseph et al., 2025; Liu Y. et al., 2025).

High-risk factors include female sex, prior cardiovascular disease such as old myocardial infarction, specific tumor types such as melanoma and thymoma, and older age (CPAEs incidence 28.7% in patients ≥65 years versus 13.5% in adults) (Joseph et al., 2025; Liu Y. et al., 2025; Cavalcante et al., 2024). Among antitumor immunotherapy regimens, combination ICI therapy, such as anti-PD-1 plus anti-CTLA-4 or anti-LAG-3, carries a significantly higher risk of myocarditis than monotherapy (adjusted OR = 4.0) (Munir et al., 2026; Wang et al., 2023); CRS induced by CAR-T therapy can directly trigger cardiac dysfunction and arrhythmias (Baker et al., 2023). CAR-NK, as an emerging therapy, currently lack large-scale clinical data, but their innate cytotoxic activity and ADCC effects may indicate that their cardiotoxicity could combine the autoimmunity of ICIs with the CRS profile of CAR-T (Wang et al., 2023; Baker et al., 2023).

### 3.2 Mechanisms of cardiotoxicity

The core mechanism of ICIs-CTRCD is disruption of immune homeostasis, including the following common pathways.

1. Excessive activation of T/NK cells and myocardial infiltration: CXCR6+/CXCR3hi CD8^+^ T cells aggregate in the heart through the CXCL16/CXCL10 axis, and hypoxia and metabolic stress in the TME may further reshape NK cell chemotaxis, prompting migration toward myocardial tissue with high CXCL9/10 expression (Barmada et al., 2023; Blum et al., 2024) and driving amplification of inflammatory-factor cascades (Munir et al., 2026; Ma et al., 2024). Subsequent CRS directly damages myocardial mitochondria and disrupts calcium homeostasis through IL-6, TNF-α, IFN-γ, and other mediators, while activating endothelial cells to express vWF and Ang-2 and inducing microvascular obstruction (Totzeck et al., 2022);
2. Autoantibodies and molecular mimicry: after exposure of myocardial-specific antigens, α-myosin-specific T cells can differentiate and proliferate, inducing myocarditis in humans (Axelrod et al., 2022). However, TCR sequencing in 28 patients with irMyocarditis showed that only a small number of clones recognized this antigen, whereas most expanded clones targeted unknown myocardial antigens (Blum et al., 2024), challenging the universality of the molecular-mimicry hypothesis;
3. Inflammatory amplification loop: in the CCR2+ macrophage-CD8^+^ T-cell positive-feedback loop (the IL-12b/TNF/IFN-γ axis), IFN-γ activates macrophages to secrete CXCL9/10, thereby recruiting more T cells, forming a positive-feedback cascade, remodeling the myocardial immune microenvironment, and making the heart more sensitive to subsequent injury. Activation of the NLRP3 inflammasome mediates GSDME-dependent pyroptosis and releases mtDNA to activate the cGAS-STING pathway, causing irreversible tissue damage (Lu et al., 2025; Sun et al., 2024; Jimenez et al., 2025).

### 3.3 Clinical risk assessment and management

Current clinical assessment relies on baseline evaluation and requires integration of medical history, function, and biomarkers. Enhanced screening should be initiated in patients with prior heart failure, coronary heart disease, or valvular disease: 12-lead ECG, high-sensitivity troponin, NT-proBNP, and echocardiography including global longitudinal strain (GLS) (Totzeck et al., 2022). NYHA class and ECOG score should be routinely collected; baseline ECG should focus on QTc and conduction abnormalities (Biersmith et al., 2020). Hs-cTnT is the most sensitive early warning marker for ICI myocarditis, elevated in 94% of confirmed patients, and cTn levels at admission, peak, and discharge are all strongly positively associated with the risk of major adverse cardiac events (MACE); patients with discharge cTnT ≥1.5 ng/mL have a fourfold increased risk of MACE (Palaskas et al., 2020). BNP/NT-proBNP has independent prognostic value for cardiac dysfunction (Biersmith et al., 2020). Dynamic monitoring emphasizes being “early, frequent, and precise”: The incidence of CV events among patients with elevated troponin levels was 55% (Totzeck et al., 2022). Therefor using the time of infusion as the baseline, conduct intensive monitoring during the acute phase (especially the first 7 days), and make individualized adjustments based on CRS status and risk stratification (Totzeck et al., 2022). What’s more, cardiovascular magnetic resonance (CMR), as the gold standard for confirmation, is especially suitable for patients with persistent late gadolinium enhancement (LGE) to assess fibrosis burden (Song, 2024).

Acute interventions follow a tiered approach (Nguyen et al., 2022; Totzeck et al., 2022). First-line treatment involves methylprednisolone (Nguyen et al., 2022; Totzeck et al., 2022). For immune checkpoint inhibitor (ICI)-associated myocarditis, if there is a poor response to glucocorticoids, second-line immunosuppressants such as abatacept (a CTLA-4-Ig fusion protein that targets CD80/CD86; peripheral monocyte CD86 receptor occupancy must be monitored to maintain a level >80%) may be added (Nguyen et al., 2022; Totzeck et al., 2022). Tocilizumab (an IL-6 receptor antagonist) is the first-line treatment for cytokine release syndrome (CRS) associated with CAR-T cell therapy; its routine use for ICI-induced myocarditis is not recommended (Nguyen et al., 2022; Totzeck et al., 2022). Novel strategies such as ruxolitinib (a JAK inhibitor) and neutralizing anti-Cxcl10 antibodies have been shown in animal models to block neutrophil infiltration (Jimenez et al., 2025; Wu et al., 2024). Long-term management requires multidisciplinary collaboration, with at least 1 year of cardiac follow-up for survivors, focusing on LVEF recovery and delayed arrhythmias (Joseph et al., 2025; Song, 2024).

## 4 Chapter 3: potential risks and knowledge gaps in the cardiotoxicity of NK cell-based immunotherapy

### 4.1 NK cell-based immunotherapy may involve indirect or potential cardiovascular injury

Existing safety-assessment systems mainly focus on grading acute CRS and monitoring neurotoxicity (Tocchetti et al., 2024; Farmanieh et al., 2026). But subclinical cardiovascular injury, chronic remodeling risk, and long-term follow-up data remain almost blank (Tocchetti et al., 2024). CAR-NK cell therapy has shown lower CRS incidence and severity in preclinical and early clinical trials because of its antigen-independent activation, limited *in vivo* expansion capacity, and lower requirement for costimulatory signals (Ma et al., 2023). However, it’s discussed in the previous chapter. Cardiotoxicity is broadly relevant across immunotherapies, and the safety advantages of NK cell-based immunotherapy should not be mistaken for absence of risk (Farmanieh et al., 2026).

1. CRS-mediated indirect cardiovascular injury

NK cells secrete significantly less IL-6 and IL-1β than activated T cells. Its CRS profile is dominated by IFN-γ, GM-CSF, and TNF-α. Furthermore, clinical manifestations are typically mild, ranging from Grade 1 to Grade 2 (Zhou et al., 2023). However, the low-grade inflammation still has clear potential for cardiovascular injury (Godinho et al., 2023). Even mild CRS can trigger capillary leak syndrome (Godinho et al., 2023). This leads to increased endothelial permeability and a decrease in effective circulating blood volume (Godinho et al., 2023). This, in turn, leads to inadequate myocardial perfusion and compensatory tachycardia, which exacerbates myocardial oxygen consumption (Godinho et al., 2023). Moreover, TNF-α can downregulate sarcoplasmic reticulum Ca2+-ATPase (SERCA2a), induce mitochondrial dysfunction, and promote imbalance between matrix metalloproteinases (MMPs) and tissue inhibitors of metalloproteinases (TIMPs). This leads to suppress myocardial contractility and promoting ventricular remodeling (Khorasanchi et al., 2022). Although IL-6 and IL-1β concentrations are lower, they may still be sufficient to induce cardiomyocyte apoptosis, expression of profibrotic factors such as TGF-β, and synergistic activation of the NLRP3 inflammasome through JAK-STAT and NF-κB pathways (Freeman and Swartz, 2020). And this leads to amplify the risk of myocardial pyroptosis (Freeman and Swartz, 2020).

In the context of solid tumors, this risk becomes further complicated (Luo et al., 2025). In TME, higher doses or more frequent infusions of NK cells are required to maintain therapeutic efficacy (Yang et al., 2024). This will prolong the duration of systemic cytokine exposure (Yang et al., 2024).

2. Potential direct cytotoxicity through the perforin/granzyme B pathway

In mouse models of acute myocardial infarction, mature NK cells massively infiltrate ischemic myocardium through a CCR2-dependent pathway on days 3–7 after infarction, and their high expression of GzmB directly induces cardiomyocyte apoptosis (Cohen et al., 2026). In viral myocarditis models, the extent of NK cell infiltration is positively correlated with the severity of myocardial injury (Ambreen et al., 2025). GzmB can enter vascular smooth muscle cells through endocytosis and induce apoptosis without perforin assistance, suggesting that it may also directly damage vascular-wall structure (Ye J. et al., 2024).

Extrapolating this mechanism to CAR-NK treatment, the theoretical risk cannot be ignored. HER2 has basal expression in normal cardiomyocytes. Although preclinical CAR-NK studies have not shown toxicity against nonmalignant HER2+ cells (Godinho et al., 2023), this conclusion is based on short-term *in vitro* co-culture and cannot simulate the long-term and dynamic immune environment *in vivo*. Moreover, the influence of CAR affinity optimization and costimulatory-domain selection on off-target effects in cardiac tissue has not yet been systematically evaluated (Joseph et al., 2025).

3. Immune crosstalk

The TME induces NK cell functional exhaustion, manifested by NKG2D downregulation, PD-1/TIM-3 upregulation, and weakened IFN-γ secretion (Zhou et al., 2023). This exhaustion is not functional silence but a shift toward a proinflammatory phenotype: NK cells retained in the peripheral circulation continuously secrete GM-CSF, which strongly drives expansion of granulocyte/macrophage progenitors (GMPs) in the bone marrow and subsequently promotes monocyte infiltration into the myocardium, ultimately amplifying local inflammation (Cohen et al., 2026). As a high-metabolism organ with low regenerative capacity, the heart is highly sensitive to inflammatory mediators. When the NLRP3 inflammasome is activated by DAMPs such as HMGB1 and mtDNA during this process, it cleaves pro-IL-1β/pro-IL-18 and triggers cardiomyocyte pyroptosis and fibrosis (Freeman and Swartz, 2020).

This leads to the following consequences: circulating factors released by the tumor itself have already caused subclinical myocardial injury; activation of systemic immune responses by NK cell therapy can aggravate myocardial inflammation; and dysbiosis of the gut microbiota, which often occurs after immunotherapy, may further worsen inflammatory injury (Song et al., 2022).

### 4.2 New destabilizing factors

While CAR-NK strategies enhance *in vivo* NK cell persistence and antitumor cytotoxicity, they may also alter the dynamic balance of immune activation, thereby introducing new safety uncertainties.

1. Off-target cytokines trigger cardiac remodeling

IL-15 and IL-21 both signal through the JAK1/JAK3-STAT5 axis, and this pathway has been shown in pressure-overload heart-failure models to directly drive fibroblast activation and type I collagen deposition (Huang R. et al., 2025). Chronic low-grade IL-15 exposure from engineered NK cells, such as CAR-NK cells persisting *in vivo* for several weeks, may induce subclinical interstitial remodeling through this pathway (Huang R. et al., 2025). On the other hand, IL-15 has been shown to enhance NK cell glycolysis (the Warburg effect), which may lead to inadequate energy supply in cardiomyocytes that depend on oxidative phosphorylation. However, the direct regulatory effect of IL-15 on the AMPK/mTOR pathway in cardiomyocytes remains unknown. Existing literature only describes its metabolic reprogramming effects in immune cells, and a coculture system using hiPSC-derived cardiomyocytes (hiPSC-CMs) and mbIL-15-NK cells has not yet been established (Huang R. et al., 2025).

2. Interference between immunotherapy and conventional medications

Conventional cardiovascular baseline medications may reduce cardiac injury caused by NK cell immunotherapy: β-blockers can theoretically weaken IL-15 signaling by inhibiting the cAMP-PKA pathway and downregulating JAK2 expression, while ACEI/ARB drugs may indirectly protect the myocardium from cytokine-induced mitochondrial injury by suppressing Ang II-mediated oxidative stress (Huang R. et al., 2025). The net effects of both medication classes in the context of CAR-NK treatment still require further investigation and confirmation (Huang R. et al., 2025).

Combination immunotherapy may also superimpose cardiotoxicity. When CAR-NK is combined with PD-1 inhibitors, IFNγ levels in MIS-C models have been shown to be strongly positively correlated with TIM3+ T-cell activation and granzyme B secretion by CD16^+^ NK cells, while IFNγ itself can induce cardiomyocyte iNOS expression and excessive NO production, aggravating contractile dysfunction (Hoste et al., 2022). Anthracycline drugs (such as doxorubicin) cause cardiac damage through mechanisms such as oxidative stress and impaired iron metabolism: chemotherapy causes mitochondrial damage, leading to the production of large amounts of reactive oxygen species (ROS) (Huang R. et al., 2025; Wang et al., 2025b). Even after chemotherapy has ended, damaged mitochondria may continue to exhibit high levels of ROS leakage over the long term (Huang R. et al., 2025; Wang et al., 2025b). It can directly damage cardiac muscle cells, leading to symptoms such as reduced cardiac function, arrhythmias, and pericarditis (Huang R. et al., 2025; Wang et al., 2025b). Autoimmune reactions and CRS triggered by immunotherapy may overlap in timing and share common mechanisms. Causing a “second-hit” to the heart (Huang R. et al., 2025; Wang et al., 2025b).

### 4.3 Knowledge gaps in current clinical research progress

At present, NK cell-based immunotherapy shows promising clinical prospects in solid tumor treatment, and its superior safety has been repeatedly verified in multiple phase I/II trials (Zhu et al., 2024; Jørgensen et al., 2025; Lin et al., 2026). However, many knowledge gaps remain regarding its cardiovascular risks.

1. Lack of clinical epidemiological data

In current clinical trials, the documentation of cardiac adverse events relies heavily on CTCAE v5.0 (Rejeski et al., 2024). This system classifies cases based solely on absolute values (such as the degree of LVEF decline or the fold increase in troponin levels) (Rejeski et al., 2024). This approach overlooks the temporal dynamics of myocardial injury, the trajectory of recovery, and the state of functional compensation, resulting in a large number of subclinical events being classified as “event-free” and thus going underreported (Rejeski et al., 2024). In the field of NK cell-based immunotherapy, no prospective, multicenter, systematic study dedicated to the cardiovascular safety of NK cell products has yet been conducted. All reports of arrhythmia, heart failure, or myocarditis are scattered across case reports or secondary endpoint analyses and lack unified definitions, standardized follow-up, and independent cardiology criteria (Marin et al., 2024). Moreover, the cardiotoxicity profiles of patients with solid tumors and hematologic malignancies are fundamentally different: solid tumor patients often have older age, underlying coronary heart disease, metabolic syndrome, and cumulative injury from radiotherapy and chemotherapy, whereas hematologic malignancy patients are often relatively younger and have better cardiac reserve (Liu J.-H. et al., 2025). Existing literature has not stratified and compared these two groups (Liu J.-H. et al., 2025).

2. Fragmented mechanistic understanding

Current mechanistic research shows the pattern of “macroscopic consensus but microscopic lack of evidence.”

At the level of direct injury, although *in vitro* experiments suggest that NK cells can induce cardiomyocyte apoptosis through the perforin/granzyme B pathway (Dieterlen et al., 2026), this finding has not been validated in any *in vivo* model, especially humanized solid tumor models. Functional reprogramming, phenotypic transition, and direct interactions between cardiac-resident NK cells and cardiomyocytes after NK cell infusion remain completely unexplored (Shalaby and Abdelalim, 2025).

Regarding indirect injury, CRS is widely considered the main mechanism of cardiotoxicity in immunotherapy, but the incidence (<12%), peak intensity (mostly grade 1-2), and cytokine profile of CRS in NK cell therapy, including weaker IL-6 dominance than in CAR-T, are all markedly different from CAR-T and other immunotherapies (Zhu et al., 2024; Jørgensen et al., 2025; Shapiro et al., 2025). No NK-specific “cardiotoxicity threshold model” has yet been constructed on this basis.

In addition, immune crosstalk between the TME and cardiac microenvironment (CME) has not been confirmed in detail. Solid tumors are often accompanied by systemic hypoxia and lactic acidosis, which may upregulate PD-L1 expression in cardiomyocytes and inhibit the NK cell mitochondrial respiratory chain (Patin et al., 2022); however, whether such metabolic disturbances promote abnormal NK cell accumulation in the heart or trigger off-target activation remains unproven.

3. Absence of long-term follow-up outcomes

At present, the median follow-up time in most NK cell trials is less than 2 years (Zhu et al., 2024; Jørgensen et al., 2025; Lin et al., 2026), whereas delayed cardiomyopathy, immune-mediated vasculitis, accelerated atherosclerosis, and similar lesions often manifest clinically years to more than a decade after treatment (Liu J.-H. et al., 2025). For example, a cross-sectional study of patients receiving long-term LVAD support found that mechanical circulatory support for ≥36 months can cause progressive exhaustion of dendritic cells, CD56^+^ NK cells, and B cells, accompanied by an increased proportion of CD57^+^ NK cells, suggesting that chronic immune remodeling may constitute a long-term cardiovascular risk (Dieterlen et al., 2026). However, whether similar mechanisms occur after NK cell infusion has not been tracked. At the same time, whether current screening strategies for cancer therapy-related cardiac dysfunction (CTRCD) are applicable to NK cell therapy remains unvalidated in terms of sensitivity and cost-effectiveness, and the preventive use of baseline cardiovascular drugs such as ACEI/ARB and β-blockers also lacks evidence from randomized controlled trials (Liu J.-H. et al., 2025).

4. Lagging monitoring and diagnostic standards

Biomarkers lack NK cell-specific cutoff values: preliminary threshold recommendations for troponin (cTn) and NT-proBNP have been proposed in CAR-T-related myocardial injury (Liu J.-H. et al., 2025), but in the CAR-NK context, the associations among the magnitude and duration of elevation and deterioration of cardiac function have not been established. The prognostic value of novel biomarkers such as sST2, Galectin-3, and miR-208a in NK cell immunotherapy remains completely unknown (Liu J.-H. et al., 2025).

Imaging assessment standards are seriously delayed: echocardiography relies on LVEF, a late-stage indicator, and has almost no ability to identify early myocardial fibrosis or microvascular dysfunction. High-sensitivity parameters such as cardiac magnetic resonance T1 values and global longitudinal strain (GLS) have shown evaluative potential in other immunotherapies (Liu J.-H. et al., 2025), but have not been formally incorporated into any NK cell trial. Molecular imaging techniques, such as 68Ga-FAPI PET, can specifically visualize activation of myocardial fibroblasts, but their application remains at the conceptual stage (Liu J.-H. et al., 2025).

5. Practical challenges in clinical translation

Although current humanized mouse models can partially simulate human NK cell function, their CME differs significantly from that of humans, and the validity of cardiotoxicity prediction is questionable (Dieterlen et al., 2026). Advanced models capable of simultaneously integrating interactions between the solid tumor TME and cardiac tissue, such as heart-tumor organoid coculture chips, remain at the conceptual stage (Liu et al., 2026). At the regulatory level, CTCAE v5.0 broadly classifies items such as “myocarditis” and “pericarditis” under “cardiac disorders,” without distinguishing immune-mediated, infectious, or other causes, and it does not include NK cell-specific phenotypes such as CD56^+^ subset exhaustion or CD16-mediated ADCC-related endothelial injury (Rejeski et al., 2024). Cardiovascular endpoints are included at an extremely low rate in NK cell clinical trials; standardized definitions and independent cardiology review processes are lacking, making safety data insufficient to support regulatory decision-making (Li et al., 2025).

## 5 Chapter 4: preliminary exploration of clinical monitoring and management strategies

### 5.1 Future-oriented clinical study design for cardiovascular-safe NK cell-based immunotherapy

The general consensus is that clinical investigation of the safety of NK cell-based immunotherapy should draw on experience from other immunotherapies, such as ICIs and CAR-T, and place risk assessment and dynamic monitoring at the core. The basic framework depends on comprehensive pretreatment evaluation of patients' baseline cardiovascular risk, including medical history, biomarkers, and cardiac imaging, as well as regular monitoring during treatment of myocardial injury markers such as troponin and BNP/NT-proBNP, electrocardiography, and cardiac function.

Regarding standardized definition and grading of cardiovascular endpoint events, the International Cardio-Oncology Society (ICOS) has proposed a pathophysiological stratification framework: immune-mediated myocardial injury is characterized by elevated cardiac troponin with CMR-confirmed LGE/ECV abnormalities, whereas cardiac dysfunction caused by hemodynamic stress presents as significantly elevated BNP/NT-proBNP with normal myocardial enzymes and often rapidly improves after tocilizumab treatment (Bao et al., 2024). Embedding MACE into protocol design from the outset and independently assessing the incidence of symptomatic heart failure can avoid missed events and attribution bias caused by vague definitions (Lei et al., 2025).

Baseline cardiovascular risk stratification must balance general applicability with the specific features of NK cell-based immunotherapy. Although the HFA-ICOS score applies to most immunotherapy populations, two aspects require strengthened investigation in the CAR-NK setting:

1. solid tumor type itself is an independent risk factor, as patients with lung cancer and gastrointestinal tumors often have chronic hypoxia, malnutrition, and systemic inflammation, significantly amplifying the compensatory myocardial load caused by immune activation (Wang F. et al., 2025);
2. CHIP, as an emerging biomarker, makes carriers more prone to IL-6-driven endothelial activation and microthrombosis after immune stimulation, and CAR-T studies have already shown its association with early deterioration of cardiac function (Bao et al., 2024). In addition, baseline ferritin and IL-6 levels have been validated as strong predictors of CRS-related cardiac dysfunction and should be included in routine screening (Li et al., 2024).

Dynamic monitoring should integrate “biomarkers-imaging-cardiac functional testing” as a triad. High-sensitivity troponin (hs-cTnT), Because of its high sensitivity, should be the preferred marker for excluding acute myocardial injury (Lehmann et al., 2023). Cardiac magnetic resonance (CMR), although not a first-line screening tool, also has irreplaceable value for confirming suspected immune-related myocarditis (Biersmith et al., 2020).

In addition, miR-145 is negatively correlated with IL-6 and TNF-α, making it a relatively sensitive diagnostic biomarker (Wu et al., 2020). miR-145 also helps balance the inflammatory response and exerts a cardioprotective effect (Wu et al., 2020). The feasibility of incorporating miR-145 as a diagnostic marker should be considered (Wu et al., 2020).

### 5.2 Optimization of clinical treatment and management through interdisciplinary collaboration

Cardiovascular safety management for NK cell-based immunotherapy requires deep multidisciplinary collaboration centered on a cardio-oncology team, whose core functions cover the entire cycle of pretreatment optimization, real-time decision-making during treatment, and long-term follow-up after treatment (Bao et al., 2024).

Practical implementation depends on team-based risk communication and shared decision-making. For patients with prior cardiomyopathy or LVEF <50%, benefit-risk quantification should be performed using individualized risk models that integrate CHIP status, inflammatory markers, and tumor burden, and expected outcomes of different treatment pathways should be presented using visualization tools (Bao et al., 2024).

Management of acute events requires differentiated consideration. For confirmed immune-related myocarditis, pulse therapy with methylprednisolone is recommended), which followed by MMF maintenance (Nguyen et al., 2022). For cardiac dysfunction associated with CRS, the use of tocilizumab should be standardized to avoid excessive suppression of antitumor immunity (Tocchetti et al., 2024).

### 5.3 Medical ethical considerations and integration of patient-reported outcomes

Deeper cardiovascular monitoring inevitably brings ethical tension: the economic burden, psychological anxiety, and privacy risks caused by frequent biomarker testing and CMR examinations require researchers to seek a balance between scientific rigor and patient wellbeing (Bao et al., 2024). Elevating cardiovascular safety to a core dimension of efficacy evaluation not only aligns with patient-centered medical ethics but can also drive developers to optimize NK cell product design at the source (Lei et al., 2025).

## 6 Conclusion

Among immunotherapy strategies for solid tumors, NK cell-based immunotherapy is showing considerable advantages. These include non-HLA restriction, potential for off-the-shelf supply, low risks of CRS/ICANS and GvHD, and stronger metabolic tolerance and chemotactic infiltration than T cells in the TME. At present, strategies such as genetic engineering, multispecific antibody engagement, and cytokine/metabolic reprogramming already have preclinical and early clinical evidence supporting their effectiveness in reversing TME-induced NK cell exhaustion and restoring NK cell cytotoxicity. CAR-NK is expected to become a solid tumor immunotherapy strategy superior to CAR-T.

However, although research on various enhancement strategies continues to advance, systematic study of their cardiovascular safety remains absent, and clinical data are scarce. The median follow-up time in existing phase I/II trials is less than 2 years, and prospective cardiovascular endpoint design, NK cell-based immunotherapy specific standardized biomarker cutoff values, and human heart-tumor interaction models are lacking. As a result, events such as subclinical myocardial injury, delayed cardiac dysfunction, and immune-mediated myocarditis are almost in monitoring blind spots. Mechanisms such as CRS, the perforin/granzyme B pathway, immune crosstalk, and chronic cytokine exposure could theoretically damage the cardiovascular system through indirect inflammatory injury, direct cytotoxicity, and myocardial remodeling, respectively. However, current understanding remains more risk inference than empirical evidence, and more specific and systematic clinical studies are needed to fill these knowledge gaps.

In the future transition of NK cell-based immunotherapy from theory to clinical practice, the more mature clinical management experience of prior immunotherapies such as ICIs and CAR-T can be used as a reference. With comprehensive risk assessment and dynamic monitoring as the core, systematic clinical validation should be carried out to gradually define treatment and management standards for NK cell-based immunotherapy and promote its transformation from theory to real-world application. As research on NK cell-based immunotherapy continues to deepen, it is expected to become a more efficient, safer, shorter-cycle, and lower-cost new treatment option in the field of solid tumor therapy.

## References

[B1] Ambreen S. McCarthy A. Hidalgo A. Adrover J. M. (2025). Heart of the matter: neutrophils, cancer, and cardiovascular disease. J. Exp. Med. 222, e20242402. 10.1084/jem.20242402 40788286

[B2] Axelrod M. L. Meijers W. C. Screever E. M. Qin J. Carroll M. G. Sun X. (2022). T cells specific for α-myosin drive immunotherapy-related myocarditis. Nature 611, 818–826. 10.1038/s41586-022-05432-3 36385524 PMC9930174

[B3] Blum S. M. Zlotoff D. A. Smith N. P. Kernin I. J. Ramesh S. Zubiri L. (2024). Immune responses in checkpoint Myocarditis across heart, blood, and tumor. Nature, 636 (8041), 215–223. 10.1101/2023.09.15.557794 39506125 PMC12952943

[B4] Baker D. J. Arany Z. Baur J. A. Epstein J. A. June C. H. (2023). CAR T therapy beyond cancer: the evolution of a living drug. Nature 619, 707–715. 10.1038/s41586-023-06243-w 37495877 PMC12522170

[B5] Bao R. Hutson A. Madabhushi A. Jonsson V. D. Rosario S. R. Barnholtz-Sloan J. S. (2024). Ten challenges and opportunities in computational immuno-oncology. J. Immunother. Cancer 12, e009721. 10.1136/jitc-2024-009721 39461879 PMC11529678

[B6] Barmada A. Klein J. Ramaswamy A. Brodsky N. N. Jaycox J. R. Sheikha H. (2023). Cytokinopathy with aberrant cytotoxic lymphocytes and profibrotic myeloid response in SARS-CoV-2 mRNA vaccine–associated myocarditis. Sci. Immunol. 8, eadh3455. 10.1126/sciimmunol.adh3455 37146127 PMC10468758

[B7] Biederstädt A. Basar R. Park J. M. Uprety N. Shrestha R. Reyes Silva F. (2025). Genome-wide CRISPR screens identify critical targets to enhance CAR-NK cell antitumor potency. Cancer Cell 43, 2069–2088. 10.1016/j.ccell.2025.07.021 40845844 PMC12396527

[B8] Biersmith M. A. Tong M. S. Guha A. Simonetti O. P. Addison D. (2020). Multimodality Cardiac imaging in the era of emerging cancer therapies. JAHA 9, e013755. 10.1161/JAHA.119.013755 31960741 PMC7033826

[B9] Bray F. Laversanne M. Sung H. Ferlay J. Siegel R. L. Soerjomataram I. (2024). Global cancer statistics 2022: GLOBOCAN estimates of incidence and mortality worldwide for 36 cancers in 185 countries. CA A Cancer J. Clin. 74, 229–263. 10.3322/caac.21834 38572751

[B10] Campbell K. S. Hasegawa J. (2013). Natural killer cell biology: an update and future directions. J. Allergy Clin. Immunol. 132, 536–544. 10.1016/j.jaci.2013.07.006 23906377 PMC3775709

[B11] Cavalcante L. Chandana S. Lakhani N. Enstrom A. LeBlanc H. Schmalz J. (2024). Case report of fatal immune-mediated myocarditis following treatment with davoceticept (ALPN-202), a PD-L1-dependent CD28 costimulator and dual PD-L1/CTLA-4 checkpoint inhibitor, in combination with pembrolizumab. J. Immunother. Cancer 12, e009475. 10.1136/jitc-2024-009475 39142718 PMC11337706

[B12] Cohen R. Duval V. Al-Rifai R. Maryam S. Santos-Zas I. Braik R. (2026). NK cells promote cardiac cell death and regulate myelopoiesis in myocardial infarction. Nat. Commun. 17, 4699. 10.1038/s41467-026-71334-x 41922374 PMC13212716

[B13] Dagher O. K. Posey A. D. (2023). Forks in the road for CAR T and CAR NK cell cancer therapies. Nat. Immunol. 24, 1994–2007. 10.1038/s41590-023-01659-y 38012406 PMC12798859

[B14] Delconte R. B. Owyong M. Santosa E. K. Srpan K. Sheppard S. McGuire T. J. (2024). Fasting reshapes tissue-specific niches to improve NK cell-mediated anti-tumor immunity. Immunity 57, 1923–1938.e7. 10.1016/j.immuni.2024.05.021 38878769 PMC11684419

[B15] Dieterlen M.-T. Sauskat M. Klaeske K. Messer E. K. Jozwiak-Nozdrzykowska J. Dashkevich A. (2026). Long-term left ventricular assist device support reinforces detrimental immunological effects. Front. Immunol. 17, 1705980. 10.3389/fimmu.2026.1705980 41737206 PMC12926156

[B16] Dizaji Asl K. Velaei K. Rafat A. Tayefi Nasrabadi H. Movassaghpour A. A. Mahdavi M. (2021). The role of KIR positive NK cells in diseases and its importance in clinical intervention. Int. Immunopharmacol. 92, 107361. 10.1016/j.intimp.2020.107361 33429335

[B17] Farmanieh A. Jalali Ara A. Rashki Ghalehno S. Malek P. Rostami M. Amiri S. S. (2026). Dysbiosis of the oral microbiome and cardiovascular risk; from endothelial dysfunction to systemic inflammation. Immunopathol. Persa 12, e44026. 10.34172/ipp.2026.44026

[B18] Freeman T. L. Swartz T. H. (2020). Targeting the NLRP3 inflammasome in severe COVID-19. Front. Immunol. 11, 1518. 10.3389/fimmu.2020.01518 32655582 PMC7324760

[B19] Godinho R. Noto A. Fenwick C. Stravodimou A. Hugelshofer S. Peters S. (2023). Cytokine storm complicated by cardiogenic shock induced by anti-HER2 therapies. J. Immunother. Cancer 11, e006942. 10.1136/jitc-2023-006942 PMC10410976 37380369

[B20] Hoste L. Roels L. Naesens L. Bosteels V. Vanhee S. Dupont S. (2022). TIM3+ *TRBV11-2* T cells and IFNγ signature in patrolling monocytes and CD16+ NK cells delineate MIS-C. J. Exp. Med. 219, e20211381. 10.1084/jem.20211381 34914824 PMC8685281

[B21] Huang Y. Xie Y. Zhang Y. Liu Z. Jiang W. Ye Y. (2025a). Single-cell transcriptome reveals the reprogramming of immune microenvironment during the transition from MASH to HCC. Mol. Cancer 24, 177. 10.1186/s12943-025-02370-2 PMC12153197 40500691

[B22] Huang R. Wang X. Yan H. Tan X. Ma Y. Wang M. (2025b). Safety and efficacy of CD33-targeted CAR-NK cell therapy for relapsed/refractory AML: preclinical evaluation and phase I trial. Exp. Hematol. Oncol. 14, 1. 10.1186/s40164-024-00592-6 PMC11694373 39748428

[B23] Jimenez J. Amrute J. Ma P. Wang X. Das S. Dai R. (2025). The immune checkpoint regulator CD40 potentiates myocardial inflammation. Nat. Cardiovasc Res. 4, 458–472. 10.1038/s44161-025-00633-1 40217124 PMC12641230

[B24] Jørgensen L. V. Christensen E. B. Barnkob M. B. Barington T. (2025). The clinical landscape of CAR NK cells. Exp. Hematol. Oncol. 14, 46. 10.1186/s40164-025-00633-8 40149002 PMC11951618

[B25] Joseph T. Sanchez J. Abbasi A. Zhang L. Sica R. A. Duong T. Q. (2025). Cardiotoxic effects following CAR-T cell therapy: a literature review. Curr. Oncol. Rep. 27, 135–147. 10.1007/s11912-024-01634-2 39836349 PMC11861112

[B26] Kang X. (2025). Nanotechnology and Natural Killer Cell Immunotherapy: Synergistic Approaches for Precise Immune System Adjustment and Targeted Cancer Treatment in Gastrointestinal Tumors. 10.3389/fmed.2025.1647737 PMC12408553 40917849

[B27] Khorasanchi A. Ansari A. M. Bottinor W. Simmons G. Abbate A. Toor A. A. (2022). Transient left ventricular dysfunction following chimeric antigen receptor t‐cell‐mediated encephalopathy: a form of stress cardiomyopathy. eJHaem 3, 231–234. 10.1002/jha2.369 35846197 PMC9175695

[B28] Laskowski T. J. Biederstädt A. Rezvani K. (2022). Natural killer cells in antitumour adoptive cell immunotherapy. Nat. Rev. Cancer 22, 557–575. 10.1038/s41568-022-00491-0 35879429 PMC9309992

[B29] Lehmann L. H. Heckmann M. B. Bailly G. Finke D. Procureur A. Power J. R. (2023). Cardiomuscular biomarkers in the diagnosis and prognostication of immune checkpoint inhibitor Myocarditis. Circulation 148, 473–486. 10.1161/circulationaha.123.062405 37317858 PMC10527069

[B30] Lei W. Liu H. Deng W. Chen W. Liang Y. Gao W. (2025). Safety and feasibility of 4-1BB co-stimulated CD19-specific CAR-NK cell therapy in refractory/relapsed large B cell lymphoma: a phase 1 trial. Nat. Cancer 6, 786–800. 10.1038/s43018-025-00940-3 40251398 PMC12122374

[B31] Li J. Chen H. Xu C. Hu M. Li J. Chang W. (2024). Systemic toxicity of CAR-T therapy and potential monitoring indicators for toxicity prevention. Front. Immunol. 15, 1422591. 10.3389/fimmu.2024.1422591 39253080 PMC11381299

[B32] Li Y.-R. Zhu Y. Fang Y. Lyu Z. Yang L. (2025). Emerging trends in clinical allogeneic CAR cell therapy. Med 6, 100677. 10.1016/j.medj.2025.100677 40367950

[B33] Li D. Fu Z. Liu R. Yang F. Zhang T. Ma L. (2026). Immune microenvironment and its influence on CAR-T treatment outcomes in B-NHL. Discov. Onc 17, 1019. 10.1007/s12672-026-05084-z PMC13365089 42159874

[B34] Lin P. Acharya S. Reyes-Silva F. Basar R. Uprety N. Moreno Rueda L. Y. (2026). CD70-Targeting CAR NK cells overcome BCMA downregulation and improve survival in high-risk multiple myeloma models. Blood Cancer Discov. 7, 234–249. 10.1158/2643-3230.bcd-25-0130 41144785 PMC13012245

[B35] Liu F. Hidru T. H. Gao R. Lin Y. Liu Y. Fang F. (2020). Cancer patients with potential eligibility for vascular endothelial growth factor antagonists use have an increased risk for cardiovascular diseases comorbidities. J. Hypertens. 38, 426–433. 10.1097/HJH.0000000000002277 31584518 PMC7012358

[B36] Liu Y. Yuan X. Yang X. Yang B. Liu G. Xu X. (2025). Risk analysis of cardiovascular toxicity in patients with lymphoma treated with CD19 CAR T cells. J. Transl. Med. 23, 8. 10.1186/s12967-024-06035-4 39754193 PMC11699784

[B37] Liu J.-H. Liu K.-Y. Zhao X. Zhou X. Jiang Y. (2025). Cardiovascular toxicities associated with chimeric antigen receptor T-cell therapy. Front. Pharmacol. 16, 1578157. 10.3389/fphar.2025.1578157 40406483 PMC12094984

[B38] Liu W. Miao H. Weng L. Cao X. (2026). CAR-NK cell therapy for hematologic malignancies: advances, challenges and optimization strategies. Mol. Cancer 25, 123. 10.1186/s12943-026-02648-z PMC13141645 41882710

[B39] Lu Y. Gao J. Hou Y. Yang H. Wang D. Zhang G. (2025). Targeting the NLRP3 inflammasome abrogates cardiotoxicity of immune checkpoint blockers. J. Immunother. Cancer 13, e010127. 10.1136/jitc-2024-010127 39773567 PMC11749606

[B40] Luo N. Chen C. Zhou W. Hao J. He S. Liu Y. (2025). Natural killer cell-mediated antitumor immunity: molecular mechanisms and clinical applications. MedComm 6, e70387. 10.1002/mco2.70387 40959206 PMC12433904

[B41] Ma R. Li Z. Chiocca E. A. Caligiuri M. A. Yu J. (2023). The emerging field of oncolytic virus-based cancer immunotherapy. Trends Cancer 9, 122–139. 10.1016/j.trecan.2022.10.003 36402738 PMC9877109

[B42] Ma P. Liu J. Qin J. Lai L. Heo G. S. Luehmann H. (2024). Expansion of pathogenic cardiac macrophages in immune checkpoint inhibitor Myocarditis. Circulation 149, 48–66. 10.1161/circulationaha.122.062551 37746718 PMC11323830

[B43] Maalej K. M. Merhi M. Inchakalody V. P. Mestiri S. Alam M. Maccalli C. (2023). CAR-cell therapy in the era of solid tumor treatment: current challenges and emerging therapeutic advances. Mol. Cancer 22, 20. 10.1186/s12943-023-01723-z 36717905 PMC9885707

[B44] Marin D. Li Y. Basar R. Rafei H. Daher M. Dou J. (2024). Safety, efficacy and determinants of response of allogeneic CD19-specific CAR-NK cells in CD19+ B cell tumors: a phase 1/2 trial. Nat. Med. 30, 772–784. 10.1038/s41591-023-02785-8 38238616 PMC10957466

[B45] Munir A. Z. Gutierrez A. Krawiec C. J. Manandhar P. Shyani A. C. Ma P. (2026). CXCR6^+^ T cells drive immune checkpoint inhibitor Myocarditis. Circulation 153, 754–768. 10.1161/circulationaha.125.076976 41498147 PMC12818167

[B46] Nguyen L. S. Bretagne M. Arrondeau J. Zahr N. Ederhy S. Abbar B. (2022). Reversal of immune-checkpoint inhibitor fulminant myocarditis using personalized-dose-adjusted abatacept and ruxolitinib: proof of concept. J. Immunother. Cancer 10, e004699. 10.1136/jitc-2022-004699 35383117 PMC8984056

[B47] Palaskas N. Lopez‐Mattei J. Durand J. B. Iliescu C. Deswal A. (2020). Immune checkpoint inhibitor Myocarditis: pathophysiological characteristics, diagnosis, and treatment. JAHA 9, e013757. 10.1161/JAHA.119.013757 31960755 PMC7033840

[B48] Pan K. Farrukh H. Chittepu V. C. S. R. Xu H. Pan C. x. Zhu Z. (2022). CAR race to cancer immunotherapy: from CAR T, CAR NK to CAR macrophage therapy. J. Exp. Clin. Cancer Res. 41, 119. 10.1186/s13046-022-02327-z 35361234 PMC8969382

[B49] Pascual-Pasto G. McIntyre B. Hines M. G. Giudice A. M. Garcia-Gerique L. Hoffmann J. (2024). CAR T-cell-mediated delivery of bispecific innate immune cell engagers for neuroblastoma. Nat. Commun. 15, 7141. 10.1038/s41467-024-51337-2 PMC11336212 39164224

[B50] Patin E. C. Dillon M. T. Nenclares P. Grove L. Soliman H. Leslie I. (2022). Harnessing radiotherapy-induced NK-cell activity by combining DNA damage–response inhibition and immune checkpoint blockade. J. Immunother. Cancer 10, e004306. 10.1136/jitc-2021-004306 35314434 PMC8938703

[B51] Patone M. Mei X. W. Handunnetthi L. Dixon S. Zaccardi F. Shankar-Hari M. (2022). Risks of myocarditis, pericarditis, and cardiac arrhythmias associated with COVID-19 vaccination or SARS-CoV-2 infection. Nat. Med. 28, 410–422. 10.1038/s41591-021-01630-0 34907393 PMC8863574

[B52] Peng L. Sferruzza G. Yang L. Zhou L. Chen S. (2024). CAR-T and CAR-NK as cellular cancer immunotherapy for solid tumors. Cell Mol. Immunol. 21, 1089–1108. 10.1038/s41423-024-01207-0 39134804 PMC11442786

[B53] Pu J. Liu T. Sharma A. Jiang L. Wei F. Ren X. (2024). Advances in adoptive cellular immunotherapy and therapeutic breakthroughs in multiple myeloma. Exp. Hematol. Oncol. 13, 105. 10.1186/s40164-024-00576-6 39468695 PMC11514856

[B54] Ran G. H. Lin Y. q. Tian L. Zhang T. Yan D. m. Yu J. h. (2022). Natural killer cell homing and trafficking in tissues and tumors: from biology to application. Sig Transduct. Target Ther. 7, 205. 10.1038/s41392-022-01058-z PMC9243142 35768424

[B55] Rejeski K. Wang Y. Hansen D. K. Iacoboni G. Bachy E. Bansal R. (2024). Applying the EHA/EBMT grading for ICAHT after CAR-T: comparative incidence and association with infections and mortality. Blood Adv. 8, 1857–1868. 10.1182/bloodadvances.2023011767 38181508 PMC11007437

[B56] Shalaby K. E. Abdelalim E. M. (2025). Hypoimmune stem cells and islets: hype or a true breakthrough in diabetes treatment? Cell Mol. Biol. Lett. 30, 112. 10.1186/s11658-025-00786-8 41039199 PMC12492583

[B57] Shapiro R. M. Sheffer M. Booker M. A. Tolstorukov M. Y. Birch G. C. Sade-Feldman M. (2025). First-in-human evaluation of memory-like NK cells with an IL-15 super-agonist and CTLA-4 blockade in advanced head and neck cancer. J. Hematol. Oncol. 18, 17. 10.1186/s13045-025-01669-3 39948608 PMC11827236

[B58] Song E. J. Joachimbauer A. Tasca S. (2024). T Cells in Acute and Chronic Myocarditis: From Diagnosis to Treatment. Eur Heart J. 47 (19), 2255–2270. 10.1093/eurheartj/ehaf1080 PMC13183180 41605248

[B59] Song N.-J. Allen C. Vilgelm A. E. Riesenberg B. P. Weller K. P. Reynolds K. (2022). Treatment with soluble CD24 attenuates COVID-19-associated systemic immunopathology. J. Hematol. Oncol. 15, 5. 10.1186/s13045-021-01222-y 35012610 PMC8744064

[B60] Sun S.-J. Jiao X. D. Chen Z. G. Cao Q. Zhu J. H. Shen Q. R. (2024). Gasdermin-E-mediated pyroptosis drives immune checkpoint inhibitor-associated myocarditis *via* cGAS-STING activation. Nat. Commun. 15, 6640. 10.1038/s41467-024-50996-5 PMC11300882 39103324

[B61] Tapia-Galisteo A. Álvarez-Vallina L. Sanz L. (2023). Bi- and trispecific immune cell engagers for immunotherapy of hematological malignancies. J. Hematol. Oncol. 16, 83. 10.1186/s13045-023-01482-w 37501154 PMC10373336

[B62] Tocchetti C. G. Farmakis D. Koop Y. Andres M. S. Couch L. S. Formisano L. (2024). Cardiovascular toxicities of immune therapies for cancer – a scientific statement of the Heart Failure Association (HFA) of the ESC and the ESC Council of cardio-oncology. Eur. J. Heart Fail. 26, 2055–2076. 10.1002/ejhf.3340 39087551

[B63] Totzeck M. Michel L. Lin Y. Herrmann J. Rassaf T. (2022). Cardiotoxicity from chimeric antigen receptor-T cell therapy for advanced malignancies. Eur. Heart J. 43, 1928–1940. 10.1093/eurheartj/ehac106 35257157 PMC9123242

[B64] Wang Y. Yan K. Guo Y. Lu Y. Su H. Li H. (2023). IP-score correlated to endogenous tumour antigen peptide processing: a candidate clinical response score algorithm of immune checkpoint inhibitors therapy in multiple cohorts. Front. Immunol. 13, 1085491. 10.3389/fimmu.2022.1085491 36700205 PMC9868931

[B65] Wang D. Dou L. Sui L. Xue Y. Xu S. (2024). Natural killer cells in cancer immunotherapy. MedComm 5, e626. 10.1002/mco2.626 38882209 PMC11179524

[B66] Wang Y. Chen Y. Wang J. (2025a). Role of tumor microenvironment in prostate cancer immunometabolism. Biomolecules 15, 826. 10.3390/biom15060826 PMC12190395 40563466

[B67] Wang Y. Fu X. Li H. (2025b). Mechanisms of oxidative stress-induced sperm dysfunction. Front. Endocrinol. 16, 1520835. 10.3389/fendo.2025.1520835 PMC11835670 39974821

[B68] Wang F. Chen G. Zhang Z. Yuan Y. Wang Y. Gao Y. (2025c). The Chinese Society of Clinical Oncology (CSCO): Clinical guidelines for the diagnosis and treatment of colorectal cancer, 2024 update. Cancer Commun. 45, 332–379. 10.1002/cac2.12639

[B69] Wu S. Sun H. Sun B. (2020). MicroRNA-145 is involved in endothelial cell dysfunction and acts as a promising biomarker of acute coronary syndrome. Eur. J. Med. Res. 25, 2. 10.1186/s40001-020-00403-8 32178736 PMC7076941

[B70] Wu M. Yang Y. Cai Y. Jiang S. Xiao H. Miao C. (2024). Anti‐CTLA‐4 m2a antibody exacerbates cardiac injury in Experimental Autoimmune Myocarditis mice by promoting Ccl5‐Neutrophil infiltration. Adv. Sci. 11, 2400486. 10.1002/advs.202400486 PMC11425905 38978328

[B71] Xu L. Saunders K. Huang S. P. Knutsdottir H. Martinez-Algarin K. Terrazas I. (2024). A comprehensive single-cell breast tumor atlas defines epithelial and immune heterogeneity and interactions predicting anti-PD-1 therapy response. Cell Rep. Med. 5, 101511. 10.1016/j.xcrm.2024.101511 38614094 PMC11148512

[B72] Yang M. Li T. Guo S. Song K. Gong C. Huang N. (2024). CVD phenotyping in oncologic disorders: cardio-mirnas as a potential target to improve individual outcomes in revers cardio-oncology. J. Transl. Med. 22, 50. 10.1186/s12967-023-04680-9 38216965 PMC10787510

[B73] Ye Q.-N. Zhu L. Liang J. Zhao D. K. Tian T. Y. Fan Y. N. (2024a). Orchestrating NK and T cells *via* tri-specific nano-antibodies for synergistic antitumor immunity. Nat. Commun. 15, 6211. 10.1038/s41467-024-50474-y PMC11266419 39043643

[B74] Ye J. Baer J. M. Faget D. V. Morikis V. A. Ren Q. Melam A. (2024b). Senescent CAFs mediate immunosuppression and drive breast cancer progression. Cancer Discov. 14, 1302–1323. 10.1158/2159-8290.cd-23-0426 PMC11216870 38683161

[B75] Yen M. Ren J. Liu Q. Glassman C. R. Sheahan T. P. Picton L. K. (2022). Facile discovery of surrogate cytokine agonists. Cell 185, 1414–1430.e19. 10.1016/j.cell.2022.02.025 35325595 PMC9021867

[B76] Zhou Y. Cheng L. Liu L. Li X. (2023). NK cells are never alone: crosstalk and communication in tumour microenvironments. Mol. Cancer 22, 34. 10.1186/s12943-023-01737-7 36797782 PMC9933398

[B77] Zhu X. Xue J. Jiang H. Xue D. (2024). CAR-NK cells for gastrointestinal cancer immunotherapy: from bench to bedside. Mol. Cancer 23, 237. 10.1186/s12943-024-02151-3 39443938 PMC11515662

